# Water quality for young children in Cambodia—High contamination at collection and consumption level

**DOI:** 10.1111/mcn.12744

**Published:** 2020-08-24

**Authors:** Etienne Poirot, Somphos Vicheth Som, Frank T. Wieringa, Sam Treglown, Jacques Berger, Arnaud Laillou

**Affiliations:** ^1^ Maternal, Newborn and Child Health and Nutrition Sections United Nations Children's Fund (UNICEF) Phnom Penh Cambodia; ^2^ Independent consultant Phnom Penh Cambodia; ^3^ Institute of Research for Development (IRD) UMR Nutripass IRD‐UM2‐UM1 Montpellier France

**Keywords:** Cambodia, children under 5 years of age, coliform, *E. coli*, quality, water

## Abstract

Unsafe drinking water is a leading cause of child morbidity, especially among young children in low‐income settings. Safe water consumption requires high‐quality water available at its source and at point of use. We examined the quality of drinking water at point of collection and point of use in 796 households in three provinces, in Cambodia. Microbiological testing for coliform and *Escherichia coli* contamination was conducted for samples collected. Bivariable analysis and multivariable logistic regression were used to examine associations between various factors and the deterioration in water quality (increase in the risk according coliform or *E. coli* concentration) between point of collection and point of use. Contamination with both coliforms and *E. coli* was higher at point of use than at point of collection, with contamination at point of collection to account for 76.6% (coliforms) and 46.3% (*E. coli*). Results suggest that child drinking water represents a considerable pathway for the ingestion of pathogens, in Cambodia. Area of residence, seasonality, type of water source, and water chlorination were associated with coliform concentration between the point of collection and point of use, whereas only seasonality was associated with *E. coli* contamination (OR = 1.46; 95% CI [1.05, 2.02]). Children living in rural settings were two times more likely to drink water with a deteriorating coliform concentration between the two‐time points than children living in urban settings (OR = 2.00; 95% CI [1.22, 3.30]). The increase in coliform and *E. coli* concentrations between point of collection and point of use indicates that water contamination mostly occurs within the household. Strengthening national legislation on water quality standards and promoting safe water management at the household are needed.

**Key messages**

• The prevalence of faecal contamination in household drinking water in Cambodia is high, particularly in rural areas.

• Water contamination with coliforms and *Escherichia coli* is higher at point of use compared with point of collection.

• The major contributors to contaminated drinking water happen at the household level in Cambodia.

• Drinking water is a key pathway for ingestion of faecal and other pathogens among young Cambodian children.

• Strengthening national legislation on water quality standards and promoting safe water management at the household level are needed to reduce children's exposure to pathogens.

## 1 INTRODUCTION

In Cambodia, children under 5 years of age continue to suffer high rates of death and disability from malnutrition. Annually, approximately 4,500 deaths can be attributed to malnutrition (Moench‐Pfanner et al., 2016). This represents nearly one third of the overall child mortality rate in Cambodia. The adverse impact of malnutrition constitutes an economic burden that costs Cambodia an estimated 145 to 266 million USD annually (0.9–1.7% of gross domestic product; Moench‐Pfanner et al., 2016). Stunting, considered the most appropriate multi‐sectorial indicator for malnutrition for its nutrition sensitive and specific related, accounts for 45% of projected economic losses (Moench‐Pfanner et al., 2016). Undernutrition is both a major cause and an effect in the cycle of poverty triggered by inadequate water, sanitation and hygiene (WASH) and feeding practices (Chase & Ngure, 2016). In Cambodia, improving access to safe drinking water (65% to 83% depending on the season) and sanitation (46%), along with good hygiene practices such as hand washing (79.8%), and reducing open defecation (44%) and unsafe management of child stools (30%; National Institute of Statistics, Directorate General for Health,, & ICF International, 2015) are vital towards decreasing preventable young child deaths—especially those associated with infectious diseases such as pneumonia and diarrhoea that contribute to most of the under‐five child deaths, in Cambodia (Ministry of Health, 2017). In addition, globally, the demand for water is projected to outstrip supply by 40% in 2030 and declining water quality is becoming an issue of growing concern (Carbon Disclosure Project, 2010). Though increasing access to improved water supplies was a focus of the Millennium Development Goals (United Nations, 2015), the Sustainable Development Goals acknowledge the need to move beyond access alone and address the quality of water consumed by households and particularly by young children. Ingestion of water contaminated with faecal pathogens is a major cause of illness, disease, and environmental enteric dysfunction in young children increasing the risk of associated malnutrition and growth faltering (Tetra Tech, 2017). It is therefore important to look closer at the bacteriological quality of water to understand its potential role as a pathway of contamination and act upon it.

Many Cambodian children are given water in the first months of life as mothers believe that water intake is needed after breastfeeding (UNICEF, Helen Keller International,, & National Nutrition Program, 2016). A recent survey in Cambodia revealed that 15% of infants received water, in addition to breast milk, at 2 to 3 months of age, during the recommended period for exclusive breastfeeding (Somphos et al., 2018), and a 2016 study conducted by UNICEF in the north‐eastern region showed more than 90% of mothers gave plain water to their children aged 6 to 12 months (UNICEF, 2016). In addition, breast milk substitutes are becoming more widely used in urban areas (Pries et al., 2016), with more than 70% of children aged 6 to 12 months being bottle fed (UNICEF, 2016). Water‐borne pathogens such as cryptosporidium, amoeba, *Escherichia coli* (*E. coli*), and *Giardia duodenalis* have been associated with faltering child growth in Cambodia (Crane, Jones, & Berkley, 2015). Given the widespread provision of water to infants and young children, keeping point‐of‐use microbiological contamination low is an important measure to safeguard the nutritional and health status of Cambodian young babies and children.

Despite the early and widespread use of water as part of infant and young child feeding practices in Cambodia, there has been little local research on the source and quality of water used by children under five. This study examines the quality of household drinking water intended for consumption by children under 5 years to better understand the extent of Cambodian children's exposure to contaminated water. In doing so, our research aims to build on existing data on main household drinking water sources (National Institute of Statistics et al., 2015) and their quality (Ministry of Rural Development & World Health Organization [WHO], 2013; WHO, 2015) in Cambodia, by (a) providing a greater age‐specific focus aligned with the nutritionsensitive WASH approach proposed by Cumming and Cairncross (2016) and (b) providing a more detailed analysis of microbiological drinking water quality in line with Sustainable Development Goal 6.1 on improving access to clean water. The findings of analyses are intended to inform policies and programmes that aim to reduce water‐related risks associated with child stunting and wasting as part of broader public health strategies to reduce child undernutrition, in Cambodia.

## 2 METHODS

This study is part of a wider research project called “The Cambodian Health and Nutrition Monitoring Study” initiated by the Child Survival and Development Section from UNICEF Cambodia Country Office, in collaboration with the Institut de Recherche pour le Développement (The French research Institute for Development), the Cambodian Ministry of Health, the Cambodian Ministry of Agriculture, Forestry and Fisheries, and the Royal University of Phnom Penh. The main aim of the project is to offer feedback to the Cambodian government on the national health system and health interventions by monitoring children's health and development in selected districts of several provinces. The project was developed from a holistic view on health by including a variety of factors that influence the “physical, mental and social well‐being” of participants. These included factors related to access to health care, nutritional, access to clean water and sanitation (WASH) factors, socio‐economic situation, and cognitive development. The main interest of the collaborators of this project is child development in the first 5 years of life. This implied an observational study design with longitudinal data collection from a cohort representative of the general population selected from the six districts in three provinces (Somphos et al., 2018).

### 2.1 Study sites

Interviews with mothers of children under the age of three were conducted in Phnom Penh (Russei Kaev district), Kratie province (Chitr Borie and Krong Kratie districts), and Ratanakiri province (Ou Chum, Krong Ban Lung, and Bar Kaev districts) during two rounds of survey (the first round during the wet season and the second during the dry season) as part of a project called “MyHealth, a longitudinal study.” The main objective of the project was to collect health and nutrition monitoring data during 3 years in selected districts in three provinces.

Beside the urbanized district of Ban Lung, Ratanakiri is formed by rural settlements where most inhabitants are smallholder farmers who practice a subsistence agriculture supplemented by some food collection from surrounding forests and rivers. Ratanakiri has a large proportion of indigenous people with a high number of ethnic groups that have their own language and culture living in remote areas. The clear majority of Kratie population (80%) is ethnic Khmer (Cambodian, 80%). The Mekong river provides the population in this province the opportunity to farm and produce crops, four out of five residents are employed in agriculture, and 70% of the population in the province is concentrated along the river. Phnom Penh is the capital city of the country. District selected in Phnom Penh included (a) two districts with a good offering of health services and hospitals, as well as stable food security, and (b) one peri‐urban district, Russei Kaev with a typical, large proportion of poor urban population from mixed ethnic origins and with limited access to services. Farming is not an option in Russei Kaev, but there is an availability of various jobs for different backgrounds and multiple educational opportunities.

### 2.2 Sampling approach

A target of 139 households with children under the age of 3 years per district and per data collection round was calculated necessary based on an assumed prevalence of drinking water samples free of faecal contamination in rural areas of 22.8% (Ministry of Rural Development & WHO, 2013) and a desired impact of 4.5% per year (factoring in a 20% refusal). The list of households with children under 3 years of age from the main project “The Cambodian Health and Nutrition Monitoring Study” served as the sampling frame for sample selection process. Using random tables, the households were systematically selected. Therefore, in each district, a random subsample of 139 households was selected during each season from the sample of 5,419 households list included in the main project. Four hundred seventeen households were randomly selected during both the rainy season (October 2016) and the dry season (February 2017), leading to a total of 796 households (excluding refusals).

### 2.3 Data collection

The Multiple Indicator Cluster Survey Water Quality Module (UNICEF, 2017) was used as part of UNICEF's early childhood longitudinal study questionnaire. Two types of water samples were obtained: one collected from the source of drinking water used for children under 3 years of age and one from the main drinking vessel used by caregivers to provide water to their children aged 0–36 months (hereafter referred to as “point‐of‐collection” and “point‐of‐use” samples, respectively). For bottled water, point‐of‐collection samples were taken directly from the water bottle. Point‐of‐use samples were collected from the drinking container, or if the water was also consumed directly from the bottled water, then the point of use and point of collection were identical: at the water bottle.

Ethical approval for the study was obtained from the Cambodia National Ethics Committee for Health Research under the Ministry of Health. Participation was voluntary, and all participants provided informed consent prior to enrolment.

### 2.4 Methods for testing water samples

This analysis used the typologies of drinking water source (improved/nonimproved and piped, dug well, etc.) as recommended by WHO/UNICEF Joint Monitoring Programme for Water Supply and Sanitation (WHO/UNICEF, 2017). Microbiological analysis for human and animal faeces indicating the presence of *E. coli* and coliforms, bacteria found in soil and surface water and in human or animal waste, was conducted using the membrane filtration method (Nissui Pharmaceutical Co., Ltd., 2009). Water samples were collected in sterilized Nasco Whirl‐Pak® bags and placed immediately into cold boxes (between 1°C and 4°C) with testing conducted within less than 6 hr of storage. Samples were processed using Microfil® membrane filtration equipment and Nissui Compact Dry® plates using an electrically powered incubator with a set temperature of 37°C. Plate counts were assessed for *E. coli* and coliforms, and results were interpreted according to WHO guidelines for drinking water with the following classifications: (a) acceptable if coliform or *E. coli* counts were equal to 0 CFU/100 ml; (b) low risk if counts were 1–10 CFU/100 ml; (c) intermediate risk for 11–100 CFU/100 ml; and (d) high risk if counts were above 100 CFU/100 ml (WHO, 2011). These measurements are also in line with Cambodian drinking water quality standards that target an *E. coli* count of 0 CFU/100 ml (Ministry of Industry and Handicrafts, 2015; Ministry of Rural Development, 2015). A deterioration in microbiological water quality was defined as an increase in CFU/100 ml count to a higher concentration category between point‐of‐collection and point‐of‐use measurements.

Samples taken at the point of collection were analysed on‐site for chlorine treatment using two indicators (a) free residual and (b) total chlorine testing (Lovibond CHECKIT® Comparator), with a threshold of >0.1 mg L^−1^ used for free residual chlorine and >0 mg L^−1^ used for total chlorine in line with national standards (Ministry of Industry and Handicrafts, 2015). If total chlorine was above 0 mg L^−1^, then chlorination was used as a way to improve the quality of the water. If free residual chlorine is equal or above to 0.1 mg L^−1^, left over of chlorine is still available in the water to inactivate disease‐causing organisms. Duplicate tests were performed for ~7% of total samples for quality control, and measurement variability was lower than the predetermined 10% level of precision.

### 2.5 Statistical analysis

Chi‐square was used to assess bivariate relationships between independent variables and coliform and *E. coli* concentration dependent variables. Variables for the multivariable logistic regression model were selected through a backward stepwise conditional approach. Variables not significant in the model (*P* > 0.05) were excluded. The covariates used to build the model were area of residence (urban vs. rural), province (Phnom Penh vs. North‐East), source of drinking water (nonimproved vs. improved; WHO/UNICEF, 2017), seasonality (dry vs. wet), and presence of free residual chlorine (yes vs. no), and total chlorine (yes vs. no). Associations between those variables (area of residence, province, source of drinking water, seasonality, and presence of free residual chlorine and total chlorine) and the dependent variables of deterioration in microbiological water quality (increased risk of coliform and *E. coli*) were assessed using multivariable logistic regression models. Results are expressed as odds ratios with 95% confidence intervals. A *P* value of 0.05 was considered statistically significant. All analyses were performed using SPSS software version 20 (IBM Corp., Armonk, NY).

## 3 RESULTS

Children selected for this analysis were in majority between 6 and 24 months old (64.2%), and 28.1% were between 25 and 36 months old. Gender distribution showed homogeneous. Among the 796 water samples collected, 52.4% were collected in rural areas; 77.4% were from an improved source, defined as piped water, public tap, tube well, protected dug well, protected spring, rainwater, and (factory) bottled water (Table 1). Almost all water samples from urban settings were from an improved source (97.8%) compared with 59.1% in rural areas (P < 0.001). The consumption of bottled water represented ~30% of overall water consumption across all areas and reached 37% of water consumed in Ratanakiri.

**Table 1:** Source of drinking water by study area

	Total (%)	Phnom Penh* (%)	Kratie* (%)	Ratanakiri* (%)	Urban* (%)	Rural* (%)
Improved source of drinking water						
Piped into dwelling	25.6	64.7	14.0	0.7	51.2	2.4
Piped into yard/plot	6.2	7.8	9.1	1.8	6.9	5.5
Public tap/standpipe	0.1	0.0	0.4	0.0	0.0	0.2
Tube well or bore hole	5.7	0.0	10.2	6.5	1.8	9.1
Protected dug well	5.5	0.0	6.8	9.4	1.8	9.4
Protected spring	1.0	0.0	0.0	2.9	0.0	1.9
Rainwater	4.3	0.4	12.5	0.0	1.3	7.0
Bottled water	29.0	24.7	24.9	37.0	34.8	23.6
Nonimproved source of drinking water						
Unprotected dug well	5.3	0.0	7.2	8.3	0.0	10.1
River/dam/stream/lake/pond	5.8	0.8	10.6	5.8	0.5	10.6
Unprotected spring	8.7	0.0	0.0	25.0	0.0	16.6
Tanker truck or water vendor	2.1	0.0	3.8	2.5	1.1	3.1
Other	0.8	1.6	0.8	0.0	1.1	0.5
Total	796	255	265	276	379	415

* *P* < 0.001 (chi‐square test) for difference in prevalence between improved and nonimproved sources.

Table 2 presents the results of the microbiological tests of drinking water samples for coliform bacterial contamination. At the point of collection, ~25% of water samples did not have coliform contamination (0 CFU/100 ml). However, for samples collected at point of use, coliform concentration >100 CFU/100 ml was ~70%. Higher contamination with coliform bacteria at both point of collection and point of use was observed in rural areas, in north‐eastern provinces, for nonimproved sources, and in nonchlorinated water samples, with the prevalence of contamination at the point of collection to be significantly higher (Table 2).

**Table 2:** Coliform bacterial contamination in water samples at point of collection and point of use

	Point of collection					Point of use				
	0 CFU/100 ml (%)	1–10 CFU/100 ml (%)	11–100 CFU/100 ml (%)	>100 CFU/100 ml (%)	*P*	0 CFU/100 ml (%)	1–10 CFU/100 ml (%)	11–100 CFU/100 ml (%)	>100 CFU/100 ml (%)	*P*
Area										
Urban	45.2	11.9	11.4	31.6	0.0001	14.7	8.4	13.4	63.5	0.002
Rural	4.8	7.0	15.7	72.4		6.8	7.3	12.0	73.8	
*n*	183	72	106	413		82	61	98	535	
Season										
Wet	22.9	7.0	14.6	55.5	0.117	7.2	6.6	10.2	76.0	0.0001
Dry	24.3	11.8	12.8	51.2		14.0	9.1	15.3	61.7	
*n*	183	73	106	413		82	61	99	535	
Province										
Phnom Penh	56.5	13.3	8.1	22.2	0.0001	18.2	8.1	11.7	61.9	0.0001
North‐East	8.2	7.6	16.3	67.9		7.0	7.7	13.2	72.1	
*n*	183	73	106	413		82	61	99	535	
Source of drinking water										
Improved	29.5	11.9	15.1	43.5	0.0001	12.3	9.3	14.5	63.8	0.0001
Nonimproved	3.9	1.1	8.9	86.0		4.5	2.8	6.8	85.9	
*n*	183	73	106	413		82	61	99	535	
Main type of water										
Piped into dwelling	59.8	13.4	7.2	19.6	0.001	17.3	7.1	9.2	66.3	0.0001
Unprotected spring	0.0	1.4	13.0	85.5		0.0	0.0	4.3	95.7	
Bottled water	17.0	12.9	23.2	46.9		7.5	11.1	20.4	61.1	
*n*	154	56	75	202		51	39	67	334	
Free chlorine										
None	11.5	8.6	15.6	64.3	0.0001	8.2	7.9	13.2	70.7	0.0001
Above 0 mg L^−1^	75.5	12.9	5.4	6.1		20.5	7.5	11.0	61.0	
*n*	183	73	106	416		82	61	99	535	
Total chlorine										
Below 0.1 mg L^−1^	10.6	8.3	15.8	65.4	0.0001	8.1	7.8	13.4	70.7	0.0001
Above or equal to 0.1 mg L^−1^	73.8	13.8	5.6	6.9		20.1	8.2	10.1	61.6	
*n*	183	73	106	413		82	61	99	535	

Table 3 presents the results of the water quality test for *E. coli* bacterial contamination. At the point of collection, 53.6% of samples had no *E. coli* contamination (0 CFU/100 ml). For water intended for child consumption collected at the point of use, *E. coli* contamination >100 CFU/100 ml was 17.2%. Higher *E. coli* concentrations at point of collection were observed in water samples collected in rural areas, in north‐eastern provinces, during the dry season, from nonimproved sources, and in nonchlorinated water sources (Table 3). At the point of collection, the percentage of samples with *E. coli* concentrations >100 CFU/100 ml was 8.7% and 23.0% in urban and rural areas, respectively. At the point of use, *E. coli* contamination differed in samples by area, season, and type and source of drinking water.

**Table 3:** *Escherichia coli* contamination in water samples at point of collection and point of use

	Point of collection					Point of use				
	0 CFU/100 ml (%)	1–10 CFU/100 ml (%)	11–100 CFU/100 ml (%)	>100 CFU/100 ml (%)	*P*	0 CFU/100 ml (%)	1–10 CFU/100 ml (%)	11–100 CFU/100 ml (%)	>100 CFU/100 ml (%)	*P*
Area										
Urban	74.9	9.2	7.3	8.7	0.0001	53.9	17.1	13.8	15.2	0.0001
Rural	35.0	20.5	21.5	23.0		37.8	25.3	17.9	18.9	
*n*	411	117	114	125		353	166	124	133	
Season										
Wet	51.2	15.7	18.3	14.9	0.047	40.6	23.9	18.0	17.5	0.039
Dry	56.1	14.8	11.4	17.7		50.3	18.8	13.9	17.0	
*n*	412	117	114	125		353	166	124	134	
Province										
Phnom Penh	82.5	5.3	4.5	7.7	0.0001	52.4	17.6	14.4	15.6	0.057
North‐East	40.0	19.9	19.7	20.3		42.1	23.1	16.7	18.0	
*n*	412	117	114	125		353	166	124	134	
Source of drinking water										
Improved	63.7	12.6	13.8	10.0	0.0001	51.6	20.6	13.6	14.1	0.0001
Nonimproved	20.7	24.0	18.4	36.9		24.4	23.9	23.9	27.8	
*n*	412	117	114	125		353	166	124	134	
Main type of water										
Piped into dwelling	81.7	6.3	4.7	7.3	0.0001	49.7	17.8	16.2	16.2	0.0001
Unprotected spring	26.1	39.1	14.5	20.3		15.9	36.2	23.2	24.6	
Bottled water	70.3	14.9	8.1	6.8		59.5	20.7	10.6	9.3	
*n*	330	72	37	43		244	107	72	70	
Free chlorine										
None	44.8	18	18.1	19.1	0.0001	44.2	22.6	16.1	17.2	0.333
Above 0 mg L^−1^	91.7	3.4	0.7	4.1		50.7	16.2	15.5	17.6	
*n*	412	117	114	125		353	166	124	134	
Total chlorine										
Below 0.1 mg L^−1^	43.9	18.2	18.2	19.7	0.0001	43.8	22.7	15.9	17.5	0.221
Above or equal to 0.1 mg L^−1^	91.1	3.8	1.9	3.2		51.6	16.1	16.1	16.1	
*n*	412	117	114	125		353	166	124	134	

Water piped into the dwelling had the lowest contamination of coliform and *E. coli* at the point of collection with 59.8% and 81.7% of samples with 0 CFU/100 ml, respectively. Yet the percentage change of coliform and *E. coli* concentrations in water samples with >100 CFU/100 ml between the point of collection and point of use was the highest in samples from water piped into the dwelling with concentrations of coliform and *E. coli* at 19.6% and 7.3% at point of collection and 66.3% and 16.2% at point of use, respectively (Tables 2 and 3).

**Figure 1:**
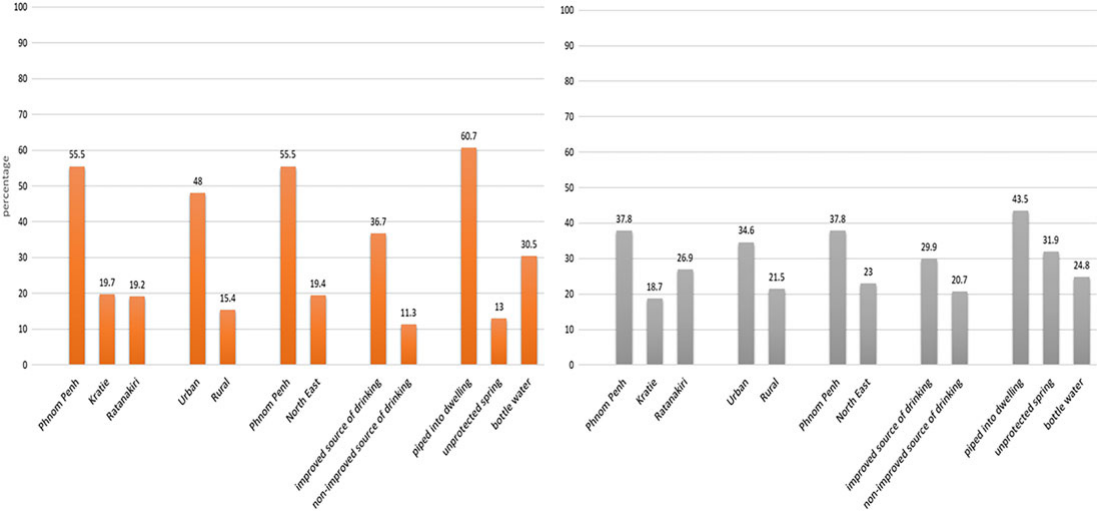
Percentage of water samples deteriorating between point of collection and point of use due to coliform (in orange) and *Escherichia coli* contamination (in grey)

The multivariable analysis for the deterioration in water quality due to coliform and *E. coli* contamination between point of collection and point of use revealed that area of residence, type of water source, and whether water was chlorine treated were associated with increased total coliform concentration between the two‐time points (Table 4, Figure 1). Children consuming water from an improved source or living in urban settings were two times more likely not to have their water deteriorating for coliforms, whereas children consuming nonchlorinated water were three times more likely to have their water deteriorating for coliforms. For the deterioration in water quality due to *E. coli* contamination between point of collection and point of use, only seasonality was associated with increased *E. coli* concentration between the two‐time points, with samples 1.5 times more likely to deteriorate during the wet season than during the dry season (Table 4, Figure 1).

**Table 4:** Factors associated with deterioration in water quality between point of collection and point of use due to coliform and *Escherichia coli* (*E. coli*) contamination

	Deterioration of total coliform			*P* value	Deterioration of *E. coli*			*P* value
	Unadjusted odds ratio	*P* value	Adjusted odds ratio		Unadjusted odds ratio	*P* value	Adjusted odds ratio	
Area								
Rural	‐	‐	‐	‐	‐	‐	‐	‐
Urban	5.06 (3.61–7.09)	0.0001	2.00 (1.22–3.30)	0.006	1.93 (1.40–2.67)	0.0001	1.33 (0.78–2.16)	0.312
Source of drinking water								
Nonimproved	‐	‐	‐	‐	‐	‐	‐	‐
Improved	4.55 (2.77–7.45)	0.0001	2.03 (1.17–3.54)	0.012	1.63 (1.09–2.45)	0.017	1.15 (0.73–1.82)	0.546
Season								
Dry	‐	‐	‐	‐	‐	‐	‐	‐
Wet	1.28 (0.94–1.74)	0.113	1.45 (1.03–2.06)	0.035	1.36 (0.99–1.87)	0.058	1.46 (1.05–2.02)	0.024
Province								
Phnom Penh	5.16 (3.71–7.19)	0.0001	1.05 (0.60–1.82)	0.876	2.04 (1.47–2.83)	0.0001	0.89 (0.96–1.72)	0.894
North‐East	‐	‐	‐	‐	‐	‐	‐	‐
Free chlorine								
None	0.11 (0.07–0.17)	0.0001	0.55 (0.19–1.66)	0.291	0.37 (0.25–0.54)	0.0001	0.75 (0.25–2.27)	0.604
Above 0 mg L^−1^	‐	‐	‐	‐	‐	‐	‐	‐
Total chlorine								
None	0.11 (0.08–0.17)	0.0001	0.33 (0.12–0.95)	0.04	0.37 (0.26–0.54)	0.0001	0.56 (0.19–1.65)	0.291
Above or equal to 0.1 mg L^−1^	‐	‐	‐	‐	‐	‐	‐	‐

Percentage of water samples deteriorating between point of collection and point of use is considerably higher in urban than in rural areas (48% vs. 15.4% and 34.6% vs. 21.5% for coliform and *E. coli*, respectively) and for improved drinking water source versus nonimproved source of drinking water (36.7% vs. 11.3% and 29.9% vs. 20.7% for coliform and *E. coli*, respectively), with water piped into the dwelling showing the highest deterioration with 60.7% and 43.5% of samples to deteriorate between the point of collection and point of use for coliform and *E. coli* concentrations, respectively.

## 4 DISCUSSION

The presence of coliform and *E. coli* bacteria in 76.6% and 46.3% of point‐of‐collection water samples, respectively, suggests that household drinking water is a significant pathway for ingestion of faecal and other pathogens by young children. Coliform contamination levels are indicative of the quality of a water supply. If coliform bacteria are present, it is likely that other microorganisms and chemical compounds are also present, further undermining children's health, growth, and cognitive development.

As approximately 59% of water samples were found to be contaminated with coliforms at the point of collection at a level above 11 CFU/100 ml in our study, including 36% of samples from improved sources having presence of *E. coli*, these findings call into question the quality of drinking water obtained from what are generally considered “improved sources.” Further, the increased coliform and *E. coli* contamination of water between point of collection and point of use suggests that an additional burden of water contamination is occurring at the household level, reinforcing the need for improved household water storage and management practices.

In Ratanakiri, water from unprotected wells was the second most common source of drinking water for children, and unfortunately, this type of source was shown to be highly contaminated with *E. coli* (74% of samples) and coliforms (100% of samples) at the point of collection. Our overall results for *E. coli* contamination show a lower prevalence than in other studies assessing the quality of household drinking water in Cambodia. Eliyan et al. in WHO (2015) found a 17% higher prevalence of *E. coli* contamination in water from urban areas (42.5%), and Ministry of Rural Development and WHO (2013) found a 12% higher *E. coli* prevalence in water samples at the point of collection (77%). As the provision of water to breastfeeding infants under 6 months is common among Cambodian mothers (Somphos et al., 2018), and any concentration of *E. coli* bacteria makes water unfit for drinking, ensuring safe household drinking water constitutes a public health priority in Cambodia.

The substantial deterioration in water quality between point of collection and point of use in our study is consistent with evidence examining these effects on preventing diarrhoeal illness in children and highlights the need to reduce water microbiological contamination at household level. This is a finding in line with a study that questioned the appropriateness of interventions to improve water quality at the point of collection for preventing diarrhoea. That limited evidence of effectiveness stressed the need to focus on household water practices (Clasen et al., 2015). Source of drinking water has shown to be a key contributor to child wasting in the north‐eastern provinces (Laillou et al., 2020) supporting the importance of water hygiene promotion in households and communities as part of a comprehensive approach for ensuring good child nutritional status.

In our study, bottled water was the most common source of household drinking water (24–37%) in the north‐eastern districts, a higher prevalence than reported in a recent national survey in which bottled water was the main source of household drinking water in 25.7% of urban, 7.7% of rural, and 10.3% of total households (National Institute of Statistics et al., 2015). The prevalence of the use of bottled water for children under five found in our study could be part of a broader growth in the use of bottled water nationally in successive CDHS datasets. Analysis indicates a total increase of the use of bottled water for drinking by almost 1.5‐fold between 2005 and 2010 and by 5‐fold between 2010 and 2014 (National Institute of Statistics et al., 2015). However, as shown in our study, local bottled water is also susceptible to contamination as almost 50% had a high concentration (>100 CFU/100 ml) of coliform contamination. Several studies on the microbiology of bottled and packed water in other regions of the world report similar violations of international quality standards (Kassenga, 2007; Obiri‐Danso, Okore‐Hanson, & Jones, 2003; Olaoye & Onilude, 2009; Oyedeji, Olutiola, & Moninuola, 2010). In addition, a recent small market survey conducted in Kratie and Ratanakiri (ILCC, UNICEF, 2017, internal report) revealed poor quality of bottled water in these areas, with counts of fungi, yeast, and heterotrophic bacteria (total count plate) above the national limit in more than 75% of water samples. These data are concerning as households presumably have confidence in the safety of bottled water and, therefore, do not treat it prior to consumption.

Our multivariable analysis seems to reinforce this point, as improved sources of drinking water were two times more likely to deteriorate for coliforms and potentially for other pathogens. Of all the water sources considered in this study, water piped into the dwelling had the lowest prevalence of faecal contamination at the point of collection. The deterioration in water quality as indicated by higher concentrations of coliform and *E. coli* at point of use indicates that most contamination of piped water into the dwelling took place within the household. Though we did not investigate the causes of household contamination, inadequate hygiene and water storage conditions are likely the major contributors. Results from drinking water surveys (WHO, 2015) have shown that most urban Cambodian households store their water before use due to frequent interruptions in water supply. More than 20% of households store water in unsealed containers and dip bowls, scoops, cups, or other vessels into the water (WHO, 2015). These findings reinforce the need for education to improve storage, handling, and treatment of water in the household and reduce the risk of pathogen exposure among household members, particularly young children, who are at a higher risk of consequent undernutrition. In Indonesia, the greatest contamination and risk factors were found in the poorest households indicating the urgent need for targeted and effective interventions there (Cronin et al., 2017). In Cambodia, a significant amount of the population is at a high risk of nonchemical contamination due to poor sanitation and shallow groundwater levels. Therefore, water quality standards as well as its enforcement mechanisms are essential for the Royal Government of Cambodia through coordinated efforts. In rural areas, integrating water quality aspects as part of the overall rural water supply monitoring system is vital, whereas in urban areas, monitoring drinking water quality standards in piped systems and supporting the licensing of private operators could reduce contamination. In the meantime, behavior change programmes that focus on household water treatment and storage practices are required with a focus on ensuring quality water for young children.

Our findings provide specific information on the extent, source, and contamination of household drinking water in distinguished locations in Cambodia. However, this research is subject to some limitations. Though data collection cycles were designed to capture humid and dry seasons, data collection for the humid season occurred during October–December 2016, with the raining season to occur between May and November, in Cambodia. Also, procedural limitations involved the use of on‐site water quality testing equipment with lower levels of precision than laboratory equipment. Finally, although measurement of *E. coli* is regarded as the most reliable measure of public health risks associated with poor quality drinking water (Wright, Gundry, & Conroy, 2004), using faecal bacteria as an indicator precludes being able to distinguish between host sources of faecal contamination, which requires a more sophisticated molecular method (Schriewer et al., 2015). To monitor the achievements in the years to come, inclusion of water quality testing at point of use in national surveys may be recommended as it is done for salt iodization in demographic health surveys for example.

## 5 CONCLUSION

Our study revealed high contamination of unimproved water sources at point of collection, as expected, and increased concentrations of faecal pathogens at point of household consumption, in water from both improved and nonimproved sources. The results highlight the importance of addressing unsafe drinking water as part of a comprehensive WASH programme to reduce faecal exposure and transmission and diarrhoeal illness to improve health and nutrition outcomes in Cambodian children. In Cambodia, increasing access to improved water sources, especially in rural areas, improving household water management and treatment practices through the adoption of good hygiene practices to prevent household‐level contamination, and furthering national and regional water quality standards for all water sources, including bottled water, are key public health priorities for Cambodia, to improve child nutritional status and development capital. These measures should be combined with community and household sanitation improvements to maximize reductions in child faecal exposure and prevent associated adverse consequences on health and nutritional status.

## CONFLICTS OF INTEREST

The authors declare that they have no conflicts of interest.

## CONTRIBUTIONS

AL, SVS, ST, and EP developed the study designed and analysed and interpreted the data. AL, JB, FW, and EP drafted the manuscript. All authors reviewed and approved the final manuscript.
